# Access and Use of Health Services by People with Diabetes from the Item Response Theory

**DOI:** 10.3390/ijerph192114612

**Published:** 2022-11-07

**Authors:** Isabela Silva Levindo de Siqueira, Rafael Alves Guimarães, Valéria Pagotto, Claci Fátima Weirich Rosso, Sandro Rogério Rodrigues Batista, Maria Alves Barbosa

**Affiliations:** 1 School of Social and Health Sciences, Pontifical Catholic University of Goiás, Avenida Universitária, número 1.440, Setor Leste Universitário, Goiânia 74605-010, Brazil; 2 Faculty of Nursing, Postgraduate Program in Nursing, Federal University of Goiás (UFG), Rua 227, Viela quadra 68, Setor Leste Universitário, Goiânia 74605-080, Brazil; 3 Institute of Tropical Pathology and Public Health, Postgraduate Program in Tropical Medicine and Public Health, Federal University of Goiás, Rua 235, Setor Leste Universitário, Goiânia 74605-050, Brazil; 4 Faculty of Medicine, Postgraduate Program in Health Sciences, Federal University of Goiás, Rua 235, Setor Leste Universitário, Goiânia 74605-050, Brazil; 5 Goiás State Health Department, Avanida SC 1, número 299, Parque Santa Cruz, Goiânia 74860-260, Brazil

**Keywords:** chronic disease, diabetes mellitus, access to health services

## Abstract

The objective of this study was to analyze the indicators of access and use of health services in people with diabetes mellitus. This study used data from the National Health Survey, conducted in Brazil in 2013. The National Health Survey was carried out with adults aged 18 years or older residing in permanent private households in Brazil. Indicators from 492 individuals with self-reported diabetes mellitus living in the Central–West region of the country were analyzed. Item response theory was used to estimate the score for access to and use of health services. Multiple linear regression was used to analyze factors associated with scores of access and use of health services by people with diabetes mellitus. The mean score of access estimated by the item response theory and use estimated was 51.4, with the lowest score of zero (lowest access and use) and the highest 100 (highest access and use). Among the indicators analyzed, 74.6% reported having received medical care in the last 12 months and 46.4% reported that the last visit occurred in primary care. Only 18.9% had their feet examined and 29.3% underwent eye examinations. Individuals of mixed-race/skin color and those residing outside capital and metropolitan regions had lower access and use scores when compared to white individuals and residents of state capitals, respectively. The study shows several gaps in the indicators of access and use of health services by people with diabetes. People of mixed race/skin color and residents outside the capitals and metropolitan regions had lower scores for access and use, suggesting the need to increase health care in these groups.

## 1. Introduction

Diabetes mellitus (DM) is a public health problem, leading to high rates of morbidity, mortality and costs for health systems and society [1]. Estimates indicate that by the year 2045, 629 million people worldwide will have DM [2].

In Brazil, the National Health Survey (Portuguese acronym: PNS) estimated the prevalence of DM self-reported and by laboratory criteria of 6.2% and 6.6% in the adult population, respectively [3]. Estimates of the Surveillance System of Risk and Protection Factors for Chronic Diseases by Telephone Survey (Portuguese acronym: Vigitel) of 2017 showed a prevalence of 7.6% of self-reported DM in adults in Brazilian capitals [4]. Between 2010 and 2013, the Global Burden of Disease (GBD) study showed that type 2 DM represented almost 5% of the disease burden in the country, with a disability-adjusted life years rate estimated at 9.2 years of life lost per thousand inhabitants [5].

DM is a sensitive condition for primary health care (PHC) [6,7], which must act as a care coordinator, providing comprehensive, resolute, and high-quality care to the population [8]. Among the attributes of the PHC, the first contact [9] stands out, defined as “gateway”, which implies accessibility and use of health services by users for each need or problem [10]. Access refers to the offer, that is, the opportunity or availability of services, and use includes all direct or indirect contact with services, such as consultations, hospitalizations, examinations, and others [11,12].

Regarding the first contact of people with DM, data from the PNS indicated that 73.2% had access to medical care, 95.3% to complementary exams, 83.3% to consultations with specialists and 57.4% to medication. Although these results reveal important access prevalence, there are still problems, considering that only 35.6% and 29.1% of the Brazilian population analyzed reported having had eye and foot exams, respectively [13].

Although these results present an overview of access indicators, based on the presence of regional differences in the access and use of health services in Brazil [14], it is important to analyze the performance of services. The analysis can be carried out through the dimensions of access and use in specific regions to identify strengths and weaknesses and contribute to the planning of care for people with DM [15]. Item response theory (IRT) enables the construction of a general score that signals the access and use of health services by individuals with DM. This is a relevant analysis for services considering the current projections of the burden of diabetes in Brazil [5] and the percentage indicator of diabetics with a request for glycated hemoglobin, which is included in the Prevent Brazil Program for monitoring the performance of PHC in the coming years [16]. Therefore, the objective of this study was to analyze the indicators of access to and use of health services in people with DM, according to sociodemographic variables.

## 2. Material and Methods

### 2.1. Data Source

Data from the PNS, developed in 2013 by the Brazilian Institute of Geography and Statistics (Portuguese acronym: IBGE) and the Ministry of Health, were analyzed. The PNS is a cross-sectional, household, population-based survey that aims to investigate the main risk and protective factors for chronic noncommunicable diseases (NCDs), as well as access to and use of health services in the adult population of Brazil [17].

The PNS was carried out with adults aged 18 years or older residing in permanent private households in Brazil. The sampling plan was by stratified clusters in three stages. The primary sampling units (PSU) were composed of census sectors of the municipalities. Secondary sampling units included permanent private households and tertiary units included adult residents of selected households [17,18,19]. The first stage of selection was performed by probability proportional to the size of the PSU subsample in each stratum, as described in previous studies [17,18,19]. The secondary selection units were selected by simple random sampling of the households of each PSU selected in the first stage. Finally, the tertiary selection units were also selected by simple random sampling among all adult residents of the selected household in the second stage [17,18,19].

The resident selected within the household in the third stage answered a specific individual questionnaire with sociodemographic data, lifestyle, NCDs, access to and use of health services, among others [19].

Data collection for the PNS was carried out between August 2013 and February 2014 [18]. Data were collected by data collection agents, trained by the Ministry of Health, and by IBGE supervisors and coordinators. The interviews were recorded on Personal Digital Assistance, also known as handheld computers [17].

### 2.2. Population

The target population of this study was adults with self-reported DM residing in the Central–West region of Brazil (Goiás, Distrito Federal, Mato Grosso and Mato Grosso do Sul) interviewed in the PNS, totaling 492 individuals.

### 2.3. Indicators of Access and Use of Health Services

Twenty-two indicators on access to and use of health services by participants with a self-reported diagnosis of DM residing in the Central–West region of Brazil were analyzed, which are listed in Table 1.

### 2.4. Independent Variables

The independent variables were: age range (18–29 years, 30–39 years, 40–59 years or ≥60 years); sex (male or female); education level (no education/incomplete elementary school, complete elementary school/incomplete high school, complete high school/incomplete higher education or complete higher education or more) [13]; self-reported race/skin color (white, black, mixed-race or others [Asian or Native American]) [21]; married/partner (no or yes); place of residence (capital, metropolitan region or other locations [outside capitals and metropolitan regions]) and federation units (Goiás, Distrito Federal, Mato Grosso and Mato Grosso do Sul).

### 2.5. Statistical Analysis

Data were analyzed using the STATA software, version 15.0, using the “survey” module for complex sampling. Initially, the sociodemographic variables and items for access and use of health services by individuals with DM were described as absolute (n), relative (%) and 95% confidence intervals (95% CI). Age as a quantitative variable was described as mean (M) and 95% CI.

Next, the IRT was used to construct a score for access and use of health services by individuals with DM. The two-parameter logistic model was used. In short, the IRT is a set of mathematical models that relate to a latent variable that cannot be observed directly but can be inferred through the analysis of variables related to it [22]. In the present study, we analyzed multiple dichotomous items (yes and no) to construct the latent variable called “access and use of health services”.

In the IRT, two parameters are generated for each item: alpha and beta. Alpha values have the following interpretation: 0 (no discrimination); 0.01 to 0.34 (very low discrimination); 0.35 to 0.64 (low discrimination); 0.65 to 1.34 (moderate discrimination); 1.35 to 1.69 (high discrimination); ≥1.70 (very high discrimination) and +∞ (perfect discrimination) [22]. The beta indicates the participant’s degree of difficulty in answering the item. Initially, the modeling was carried out with all items that presented complete data (n = 492) that encompassed 17 variables. Next, alpha values < 0.65 were excluded from the final model. The assumption of unidimensionality, assumption of the IRT, was verified through factor analysis.

We performed the IRT to create a latent variable (access and use) from several observed items to analyze the determinants of this construct from a single variable, rather than multiple variables. After the construction of the latent variable, the scores were transformed into a scale from 0 to 100, where 0 represents the lowest level of access and use and 100 the highest level.

The last stage of the analysis consisted of verifying the differences in access scores according to sociodemographic variables. In the bivariate analysis, the Mann–Whitney or Kruskal–Wallis test for independent samples was used to compare the mean scores between the variables. The effect size of this analysis was measured by Cohen’s d. In this case, the effect size was classified as negligible (d < 0.20), small (d = 0.20 to 0.49), medium (d = 0.50 to 0.79) or large (d > 0.80) [23]. Then, variables with *p*-value < 0.20 in the bivariate analysis, sex and age were included in a multiple linear regression model. The assumptions of absence of multicollinearity, linearity, absence of model specification errors and homoscedasticity were met. The regression results were presented as regression beta coefficient (β) and respective 95% CI.

In all analyses, values of *p* < 0.05 were considered statistically significant.

### 2.6. Ethical Aspects

The National Research Ethics Committee in June 2013, protocol No. 328159, approved the PNS. Participants were informed about the study and signed the Free and Informed Consent Form.

## 3. Results

### 3.1. Sample Characteristics

In the Central–West Region of Brazil, among individuals interviewed in the PNS (n = 7519), 492 (6.5%; 95% CI: 4.7–7.1) reported a medical diagnosis of DM.

Table 2 describes the characteristics of the sample. The mean age was 58.1 years (95% CI: 56.3; 58.9; minimum = 21; maximum = 94), with 49.5% of the sample aged 60 years or older. Most individuals with self-reported DM were female (61.0%), with a spouse (61.0%), with low schooling (illiterate/incomplete elementary school) (60.5%). Regarding race/skin color, 48.2% were mixed-race. Of the total, 91.3% lived in urban areas and 46.2% in locations outside capital cities and metropolitan regions.

### 3.2. Indicators of Access and Use of Health Services

Among the 22 indicators analyzed, 97.8% of people with DM underwent examinations, 74.6% reported having received medical care in the last 12 months and 46.4% reported that the last visit occurred in primary care. In addition, 18.9% had their feet and 29.3% their eyes examined in the 12 months prior to the interview. Regarding guidance, 83.8% received guidance on healthy eating and 80.7% on maintaining adequate weight (Table 3).

### 3.3. Access and Use Analysis According to Item Response Theory (IRT)

The initial IRT model comprised 17 items and the indicators that presented the highest discrimination parameters (>1.70) on the latent variable were: “received guidance on healthy eating” (α = 14.9), “received guidance on maintaining adequate weight” (α = 7.46) and “received guidance on physical activity” (α = 6.53). The items that presented the highest parameters of difficulty were: “hospitalization for DM or for some complication” (β = 4.62) and “feet examined in the last 12 months” (β = 1.38).

The items “received the last treatment for diabetes in primary care” (α = 0.49), “received the last treatment by the SUS” (α = 0.56), “hospitalization for DM or for some complication” (α = 0.40) and “have a private health plan” (α = −0.01) had a low discrimination parameter (<0.65) and were removed from the final model.

Table 4 presents the final IRT logistic model after removing items with low discrimination power. In this final model, the items “received guidance on healthy eating” (α = 17.9), “received guidance on maintaining adequate weight” (α = 8.01), “received guidance on physical activity” (α = 6.84), “received guidance on not drinking to excess” (α = 5.75), “received guidance on not smoking” (α = 5.63) and “received a request for blood tests” (α = 2.90) had the highest discrimination parameters (>1.70, very high discrimination), being the most informative items about the latent variable.

**Figure 1:**
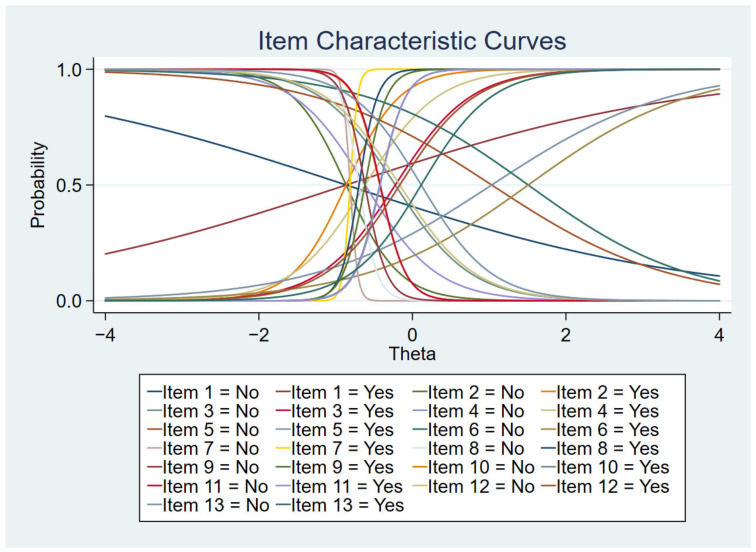
Item characteristic curves.

**Figure 2:**
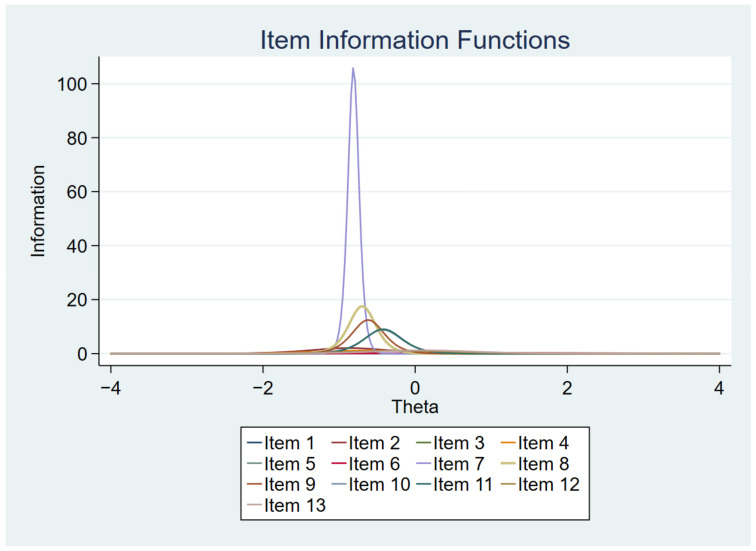
Item information functions.

**Figure 3:**
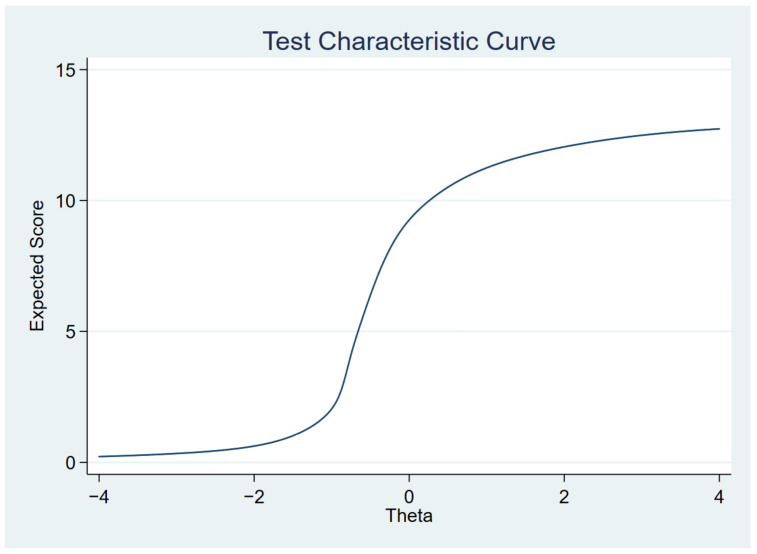
Test characteristic curve.

**Figure 4:**
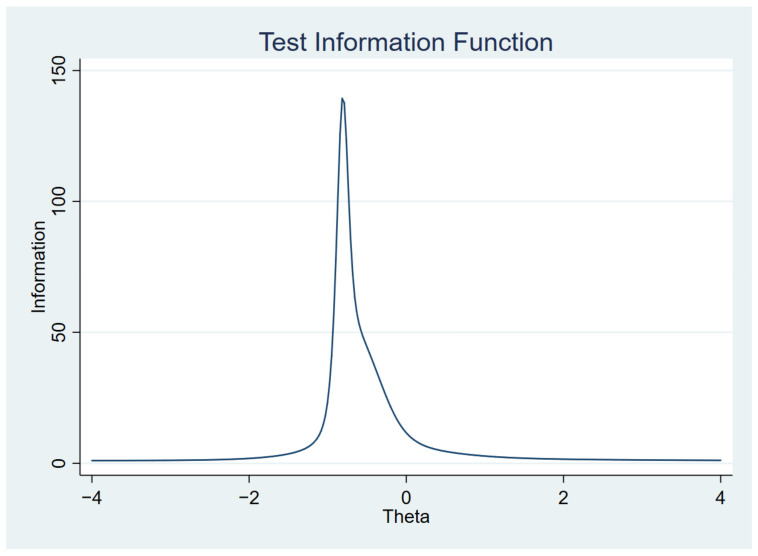
Test information function.

Figure 1, Figure 2, Figure 3 and Figure 4 show the item characteristic curves, item information functions, test characteristic curve and test information function of the final IRT model, respectively.

**Figure 5:**
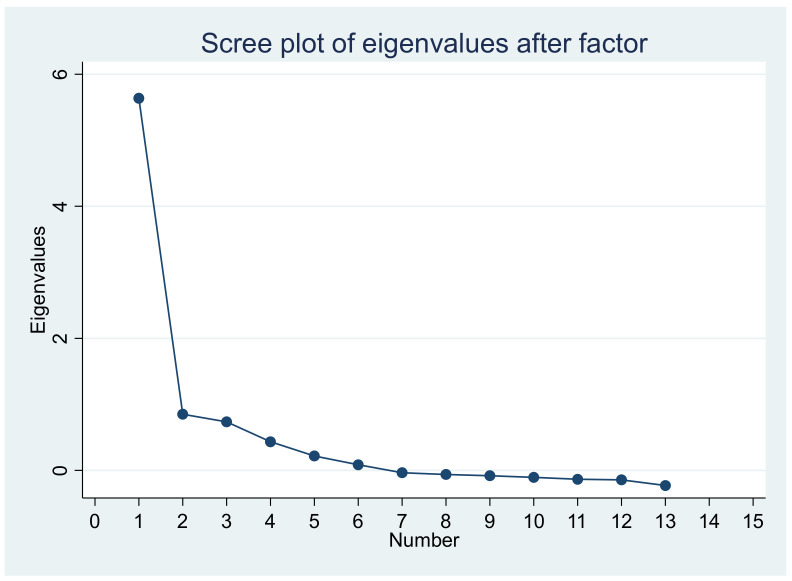
Scree plot of eigenvalues on factor analysis.

The results of the factor analysis to evaluate the unidimensionality of the model showed that the variance explained by the first factor was 78.6%, suggesting unidimensionality. Figure 5 shows the scree plot confirming the one-dimensionality.

### 3.4. Analysis of Access/Use Scores by Sociodemographic Variables

The mean access score found for the participants was 51.4 (standard error ± 1.1). Statistical differences were verified for the variable’s race/skin color (*p*-value = 0.020; d = 0.21) and place of residence (*p*-value < 0.001; d = 0.41) in the bivariate analysis. This analysis showed lower access scores in individuals of mixed race/skin color and residents outside the capitals and metropolitan regions. There was no difference in the access score according to age group, sex, education, marital status, area of residence or federation unit (*p*-value > 0.05) (Table 5).

Table 6 shows the multiple linear regression model of factors associated with scores for access to and use of health services. There was a negative association between access scores and brown race/skin color and living outside capital cities and metropolitan regions. Thus, the adjusted analysis showed that individuals of mixed-race race/color who lived outside capital cities and metropolitan regions (other regions) had statistically lower scores for access/use when compared to white individuals and residents of state capitals, respectively.

## 4. Discussion

The present study analyzed the access and use of health services by people with self-reported DM in the Central–West region of Brazil, based on the IRT. The mean access score found for the participants was 51.4 (standard deviation ± 25.2). Regarding the indicators analyzed, more than 70% of the participants received medical care for DM in the last 12 months; almost all were able to perform complementary exams; about 70% obtained medication or insulin from some SUS service and most received guidance on healthy eating, physical activity and maintenance of adequate weight. People with DM with brown skin color and residents of regions outside the capitals and metropolitan regions (other regions) had lower scores for access and use of health services.

### 4.1. Access and Use Indicators

The 22 indicators analyzed in the PNS allow an assessment of the use of health services at different points in the Health Care Network (HCN) (although not in its entirety), and the variations in some of these points deserve to be highlighted.

Regarding consultations, although more than 70% of the participants had received medical care in the last 12 months, less than half reported having had this last consultation in primary care, which was also identified in other investigations [7,13]. The Ministry of Health established protocols for the management of DM in primary care, proposing an annual medical consultation for people with diabetes [24]. It is also noteworthy that 56.6% received medical care from the same doctor they had previously consulted. The low proportion of consultations with a specialist doctor may indicate that primary care services have been able to resolve the problems of users with DM; however, they may also show weakness in the level of secondary care. This assessment can be deepened in future studies with this population.

Regarding the indicators for performing tests, both biochemical (glycated hemoglobin blood, urine) as well as eye and foot exams, almost all users with DM in the Central-West region had the requested tests performed. In addition, a high proportion had access to complementary exams, a result consistent with previous investigations [13,25]. This result is in agreement with one of the specific objectives of the Health Care Network for People with NCDs, which aims to “provide access to adequate diagnostic and therapeutic resources in a timely manner, ensuring comprehensive care” [26]. In addition, performing blood tests, such as fasting blood glucose and glycated hemoglobin, every three months and, after achieving control, every six months, and annual assessment of cholesterol (LDL and HDL) and triglycerides, aims to comply with goals of glycemic, metabolic and cardiovascular control recommended by the Ministry of Health for people with DM [24,27]. Regarding the tests recommended in the routine monitoring of people with DM, it is noteworthy that in this study the glycated hemoglobin test (HbA1c) was requested for half of the users. This corroborates other studies in which failures in the monitoring of glycemic control were pointed out due to the lack of requests for this test [28,29]. To maintain glycemic control, it is recommended to request the HbA1c test at the beginning of treatment and every three months, and it can be performed twice a year for people with DM with good metabolic control [24]. National and international studies indicate failures in ordering and performing specialized tests for people with diabetes, due to ethnic, age, geographic and health insurance coverage factors [30,31,32]. It is noteworthy that this low proportion may also be linked to the user’s lack of knowledge of the name of the exam, which may have led to a negative answer about its performance at the time of the interview.

Still in relation to exams, in this study, less than a fifth of the participants with DM had their feet examined and only 29.3% underwent an ophthalmologic evaluation in the year prior to the interview, results consistent with studies from Brazil [13,31]. On the other hand, people with DM in the United States of America who attended health services had their feet examined on average 2.7 times in a year [33]. The comparison of these studies indicates lower access for people with DM in the Central–West region of Brazil to foot exams when compared to developed countries. In Brazil, annual examination of the feet and screening for retinopathy are recommended, including an initial evaluation of the eyes at the time of diagnosis and an evaluation every year [24,34]. Therefore, these results reinforce the need to monitor these tests, as recommended, as well as reinforce the need to continuously implement preventive measures for secondary complications of DM in the scope of primary care, considering that the diagnostic and therapeutic support system is one of the transversal points of the HCN and that such preventive measures contribute to reducing future costs of health services caused by complications, such as diabetic foot amputations and blindness [35,36].

The indicators of access to medicines, whether in the Popular Pharmacy Program or another SUS service, showed a proportion of 66.5% and 70.8%, respectively, in the participants of this study. As well as the exams, they make it possible to understand another HCN support system: the pharmaceutical assistance system. The National Survey on Access, Use and Promotion of the Rational Use of Medicines (Portuguese acronym: PNAUM) showed an average physical availability of medicines of 52.9% in primary care units in a sample of 27 municipalities in the country. Moreover, in relation to oral antidiabetic drugs (metformin, glibenclamide/glicazide), it was above 80%, as recommended by the WHO; however, for insulin it was lower [37]. Access to essential medicines is one of the eight Millennium Development Goals [38]; in addition, considering the proportions found in the Central–West region of Brazil, this access can be considered low, given the increasing prevalence of DM in Brazil and the risk of its complications.

Regarding the guidelines, represented by six indicators, healthy eating and weight maintenance had the highest proportions. Subsequently, the highest proportions were for physical activity, not smoking, not drinking and measuring blood glucose. Such guidelines, associated with other PHC attributes, favor adherence to DM treatment and contribute to the individual knowing more about his/her health condition [39,40]. In addition, with the exception of blood glucose measurement, all indicators are included in the Model of Attention to Chronic Conditions (MACC) [41] as main risk factors for the prevention of chronic conditions, including Diabetes Mellitus. Considering that primary care is the main space for the development of preventive actions, these results are positive and need to be reinforced in the context of professional care.

### 4.2. Scores of Access and Use of Services

The mean access score found for the participants was 51.4 (standard error ± 1.1). An important inequality identified in this investigation was the low scores of access and use of health services that individuals with DM who declared themselves brown had, when compared to whites. Previous results from the PNS showed that some indicators of access (for example, examination of the feet and access to complementary exams) were lower in people of brown skin color [13]. Considering race/color as a proxy for socioeconomic level, these findings suggest that there are inequities of access and use of health services for brown people in the Central–West region of the country. This may be associated with income, which limits access to goods and services [42,43].

The results of the present study showed that individuals with DM who reported living outside capitals and metropolitan regions also had lower scores for access and use of health services, as already verified in a previous study [44]. This suggests that individuals residing in capitals probably have more access to health care and complementary exams than those residing in regions outside the capitals, who may possibly have the worst consequences of diabetes [45].

### 4.3. Limitations and Strengths

A limitation of this study is the cross-sectional design, which does not allow temporality between the variables analyzed and the access scores. Furthermore, other indicators of access and use of broader health services were not evaluated, such as travel to the health unit, duration of consultation, scheduling and others. Data were self-reported by participants, subject to response and memory bias. Our study also used data from 2013, so the sociodemographic characteristics of the study population may have changed over time, and changes in health service access and use scores and their associations in the Brazilian population may also have changed. Despite the limitations, this study presented an overview of the access and use of health services by people with diabetes in the states of the Central–West region of Brazil, thus pointing out that there is still a need for improvements and advances in the health system in relation to the two dimensions analyzed, including for diabetics. In addition, an IRT model was used to predict the access/use of health services in the sample under study.

## 5. Conclusions

Despite the identification of some positive results in relation to indicators of use, such as the report of guidelines on healthy eating, physical activity and maintenance of adequate weight, some indicators proved to be critical, such as the evaluation of the feet and eyes. The mean access score estimated for the participants was 51.4, indicating a medium level of access to health services by people with DM in the Central–West region of Brazil. Differences in access to and use of services were identified among mixed-race individuals and among residents of regions outside the capitals or metropolitan regions. The scores of these individuals were statistically lower when compared to white individuals living in capital cities.

## 6. Implications

Although there is already evidence on the access and use of health services by people with DM [13], this study is based on data from the PNS, which is a large sample, generalizable to the Brazilian population. This study adds to the Brazilian literature the analysis of many indicators of access and use of services, in addition to a statistical modeling that allowed the analysis of factors associated with the scores of this latent variable in people with DM. The analysis through the IRT can be replicated in national and international scenarios to analyze access to and use of health services by people with DM. Multiple indicators of access and use of health services were analyzed and gaps were identified that can improve health care for the population with DM in Brazil. In addition, the monitored indicators contribute to the monitoring of the goal of reducing the burden of NCDs such as diabetes mellitus, according to the Sustainable Development Goals, WHO Global Action Plan for the Prevention and Control of Noncommunicable Diseases (NCDs) 2013–2030 and Plano of Strategic Actions to Combat Chronic Diseases and Non-Communicable Diseases in Brazil, 2021–2030 of the Ministry of Health.

Access and use of health services are dimensions of the health system that influence glycemic control and adherence to treatment by people with diabetes [13]. Thus, this study suggests the need to reduce the inequalities still present among these individuals when they access or use a health service. In this sense, the results of this study indicate the importance of public health policies aimed at the surveillance and prevention of diabetes and the monitoring of the indicators presented, aiming at improving the performance of health services in the Central–West region that provide assistance to people with diabetes. Therefore, it is recommended to strengthen actions to qualify the primary care team in relation to what is recommended for health care for this population.

## Figures and Tables

**Table 1:** Items used in the analysis of access and use of health services.

Item	Percentage (%) of People with DM Who	Answer
1	Received medical care for diabetes in the last 12 months	Q39. When was the last time you received medical care for diabetes?
2	Received the last treatment for diabetes in primary care	Q40. Where was the last time you received medical care for diabetes? Option response: 01. Basic health unit (health center or family health unit).
3	Received the last treatment by the SUS	Q43. Was this service provided by the SUS?
4	Received medical care from the same doctor as the previous consultation	Q44. In the last consultation, was it the same doctor who attended you in the previous consultations?
5	Received a request for blood tests	Q47. Was any test requested in any of the diabetes care services? The. Blood test (cholesterol, blood glucose, triglycerides).
6	Received a request for glycated hemoglobin	Q47. Was any test requested in any of the diabetes care services? B. glycated hemoglobin.
7	Received requests for urine tests	Q47. Was any test requested in any of the diabetes care services? d. Urine analysis.
8	Performed requested exams	Q48. Did you perform all the requested exams?
9	Were referred to specialists and attended consultations with a specialist doctor	Q50. In any of the consultations for diabetes, was there a referral to a specialist doctor, such as a cardiologist, endocrinologist, nephrologist or ophthalmologist? Q51. Did you go to all consultations with a specialist doctor?
10	Eyes examined in the last 12 months	Q53. When was the last time you had an eye exam or fundus in which your pupil dilated?
11	Feet examined in the last 12 months	Q54. When was the last time a doctor or healthcare professional examined your feet for sensitivity or the presence of sores or irritations?
12	Hospitalization for DM or for some complication	Q56. Have you ever been hospitalized because of diabetes or any complications?
13	Obtained medication for DM or insulin from the Popular Pharmacy Program	Q36. Were any of the diabetes medications or insulin obtained from the Popular Pharmacy Program?
14	Obtained medication for DM or insulin obtained from a SUS service	Q37. Were any of the diabetes medications or insulin obtained from a public health service?
15	Received guidance on healthy eating	Q46. In any of the diabetes consultations, did a doctor or other health professional give you any of these recommendations? Option response: a. Maintain a healthy diet (with fruits and vegetables).
16	Received guidance on maintaining adequate weight	Q46. In any of the diabetes consultations, did a doctor or other health professional give you any of these recommendations? Option response: b. maintaining adequate weight.
17	Received guidance on physical activity	Q46. In any of the diabetes consultations, did a doctor or other health professional give you any of these recommendations? Option response: c. Practice regular physical activity.
18	Received guidance on not smoking	Q46. In any of the diabetes consultations, did a doctor or other health professional give you any of these recommendations?
19	Received guidance on not drinking to excess	Q46. In any of the diabetes consultations, did a doctor or other health professional give you any of these recommendations?
20	Received guidance on measuring blood glucose at home	Q46. In any of the diabetes consultations, did a doctor or other health professional give you any of these recommendations? Option response: g. Measure blood glucose at home.
21	Received guidance on examining their feet regularly	Q46. In any of the diabetes consultations, did a doctor or other health professional give you any of these recommendations? Option response: h. Examine your feet regularly.
22	Have a private health plan	I1. Does the participant have any private, company or public health plan (medical or dental?)

Source: [13,20].

**Table 2:** Sociodemographic characteristics of the population with self-reported DM in the Central–West region of Brazil. National Health Survey, 2013.

Variables	n	%	95% CI
Age range (years)			
18–29	12	2.	1.2–6.2
30–39	32	7.9	5.1–12.0
40–59	209	39.9	35.0–45.4
≥60	239	49.4	43.5–55.5
Sex			
Female	314	61.0	55.0–66.8
Male	178	39.0	33.2–45.1
Education level (years)			
No education/Incomplete elementary school	289	60.5	54.5–66.2
Complete elementary school/incomplete high school	61	12.3	9.0–16.6
Complete high school/incomplete higher education	84	17.1	13.2–21.9
Complete higher education or more	58	10.1	7.4–13.5
Race/skin color			
White	197	40.3	34.2–46.6
Black	53	9.6	7.1–13.0
Mixed-race	232	48.2	42.2–54.3
Others *	10	1.9	0.9–4.1
Married/partner			
No	245	39.0	33.7–44.6
Yes	247	61.0	55.4–66.3
Place of residence			
Capital	257	39.5	34.0–45.2
Metropolitan region	52	14.3	10.0–20.1
Other places	183	46.2	40.4–52.2
Federation Units			
Goiás	146	43.0	36.9–49.3
Distrito Federal	101	17.0	13.7–20.9
Mato Grosso	103	20.2	16.0–25.1
Mato Grosso do Sul	142	19.8	16.5–23.7

95% CI: Confidence Interval of 95%; * Includes Asian and Native American.

**Table 3:** Descriptive analysis of indicators of access and use of health services by people with self-reported DM in the Central–West region of Brazil. National Health Survey, 2013.

Indicators	n	%	95% CI
Received medical care for diabetes in the last 12 months	364	74.6	69.1–79.3
Received the last treatment for diabetes in primary care	228	46.4	40.6–52.4
Received the last treatment by the SUS	292	57.3	51.2–63.2
Received medical care from the same doctor as the previous consultation	271	56.6	50.4–62.6
Received a request for blood tests	393	77.7	71.2–83.2
Received a request for glycated hemoglobin	290	55.6	49.2–61.8
Received requests for urine tests	349	70.4	63.8–76.2
Performed requested exams	384	97.8	95.6–98.9
Were referred to specialists and attended consultations with a specialist doctor	69	25.7	19.4–33.1
Eyes examined in the last 12 months	158	29.3	24.5–34.7
Feet examined in the last 12 months	114	18.9	14.7–23.9
Hospitalization for DM or for some complication	68	13.5	10.2–17.7
Obtained medication for DM or insulin from the Popular Pharmacy Program	226	66.5	60.0–72.5
Obtained medication for DM or insulin obtained from a SUS service	239	70.8	63.5–77.2
Received guidance on healthy eating	414	83.8	78.9–87.2
Received guidance on maintaining adequate weight	397	80.7	75.8–84.8
Received guidance on physical activity	384	77.2	71.1–82.3
Received guidance on not smoking	348	68.9	63.1–74.2
Received guidance on not drinking to excess	347	69.4	63.6–74.6
Received guidance on measuring blood glucose at home	281	55.7	49.5–61.6
Received guidance on examining their feet regularly	236	44.5	38.8–50.5
Have a private health plan	184	36.4	30.7–42.6

95% CI: Confidence Interval; DM: Diabetes mellitus, SUS: Unified Health System.

**Table 4:** Final two-parameter logistic model of the IRT to predict access and use of health services by people with DM in the Central–West region. National Health Survey, 2013.

Items	α ^1^ (95% CI)	*p*-Value *	β ^2^ (IC95%)	*p*-Value **
Received medical care for diabetes in the last 12 months	1.69 (1.28; 2.11)	<0.001	−0.85 (−1.05; −0.64)	<0.001
Received a request for blood tests	2.90 (2.16; 3.63)	<0.001	−0.88 (−1.05; −0.71)	<0.001
Received a request for glycated hemoglobin	2.04 (1.54; 2.53)	<0.001	−0.24 (−0.38; −0.10)	<0.001
Received requests for urine tests	2.16 (1.64; 2.6)	<0.001	−0.63 (−0.79;−0.46)	<0.001
Eyes examined in the last 12 months	0.92 (0.62; 1.21)	<0.001	0.97 (0.64;1.29)	<0.001
Feet examined in the last 12 months	1.01 (0.68; 1.35)	<0.001	1.42 (1.00;1.83)	<0.001
Received guidance on healthy eating	17.9 (−0.18; 36.0)	0.052	−0.83 (−0.97;−0.68)	<0.001
Received guidance on maintaining adequate weight	8.01 (4.77; 11.2)	<0.001	−0.71 (−0.4;−0.59)	<0.001
Received guidance on physical activity	6.84 (4.35; 9.33)	<0.001	−0.63 (−0.75; −0.51)	<0.001
Received guidance on not smoking	5.63 (3.44; 7.81)	<0.001	−0.44 (−0.55; −0.33)	<0.001
Received guidance on not drinking to excess	5.75 (3.53; 7.97)	<0.001	−0.43 (−0.54; −0.33)	<0.001
Received guidance on measuring blood glucose at home	2.00 (1.51; 2.49)	<0.001	−0.18 (−0.32; −0.04)	0.009
Received guidance on examining their feet regularly	2.19 (1.65; 2.73)	<0.001	0.09 (−0.03–0.22)	0.145

^1^ Discrimination parameter: allows the item’s ability to distinguish people from different regions of the latent variable (access and use of health services); ^2^ Difficulty parameter: shows the difficulty of an item to achieve a 0.5 probability of correct answer; 95% CI: 95% Confidence Interval; * Statistically significant values (*p*-value < 0.05) for the discrimination parameter; ** Statistically significant values (*p*-value < 0.05) for the difficulty parameter.

**Table 5:** Comparison of scores for access and use of health services, according to sociodemographic variables in the Central–West region. National Health Survey, 2013.

Variables	Scores		*p*-Value	Cohen’s d
	Mean ± SE	95%CI		
Age range (years)				
21–29	50.0 (9.6)	30.9–69.0	0.970 *	0.08
30–39	51.0 (4.8)	41.6–60.3		
40–59	50.9 (1.7)	47.5–54.3		
≥60	52.0 (1.6)	48.8–55.1		
Sex				
Male	51.3 (1.4)	48.5–54.1	0.959 **	0.09
Female	51.6 (1.4)	47.9–55.3		
Education level (years)				
Complete higher education or more	50.8 (1.4)	48.1–53.4	0.186 *	0.22
Complete high school/incomplete higher education	50.3 (3.6)	43.3–57.3		
Complete elementary school/incomplete high school	51.3 (3.0)	45.3–57.1		
No education/Incomplete elementary school	55.9 (3.7)	48.6–63.2		
Race/skin color ***				
White	54.0 (1.8)	50.4–57.4	0.020 *	0.21
Black	54.6 (3.3)	48.2–61.1		
Mixed race	48.4 (1.6)	45.2–51.6		
Place of residence				
Capital	55.6 (1.6)	52.4–58.8	<0.001 *	0.41
Metropolitan region	50.3 (2.8)	44.7–55.9		
Other places	45.9 (1.8)	42.4–49.4		
Total	52.3 (1.1)	50.2–54.5		

* Kruskal-Wallis test; ** Mann-Whitney test; *** The category other than race/color was removed from the bivariate analysis due to the small number of observations; SE = standard error; 95% CI: Confidence Interval of 95%.

**Table 6:** Multiple linear regression model of factors associated with scores for access and use of health services in the Central–West region. National Health Survey, 2013.

Variables	*β*	95% CI	*p*-Value
Age range (years)			
21–29 ^®^			
30–39	1.04	−15.83; 17.92	0.903
40–59	1.66	−13.08; 16.42	0.824
≥60	2.45	−12.35; 17.25	0.745
Sex			
Male ^®^			
Female	−0.53	−5.20; 4.13	0.823
Education level (years)			
Complete higher education or more^®^			
Complete high school/incomplete higher education	−3.89	−12.41; 4.63	0.371
Complete elementary school/incomplete high school	−3.29	−12.43; 5.84	0.479
No education/Incomplete elementary school	−0.56	−8.26; 7.12	0.884
Race/color (self-declared)			
White ^®^			
Black	−0.01	−7.62; −7.60	0.997
Mixed-race	−4.97	−9.77; −0.18	0.042
Place of residence			
Capital ^®^			
Metropolitan region	−5.32	−12.93; 2.27	0.169
Other places	−9.71	−14.78; −4.63	<0.001

*β* = Regression coefficient; EP = Standard Error; 95% CI: 95% Confidence Interval; ^®^: Reference category.

## Data Availability

The microdata of this study can be accessed at the following link: https://www.ibge.gov.br/estatisticas/sociais/saude/29540-2013-pesquisa-nacional-de-saude.html?=&t=microdados (accessed on 25 August 2022).
